# Efficacy of abatacept in juvenile idiopathic arthritis related uveitis

**DOI:** 10.1186/1546-0096-9-S1-P100

**Published:** 2011-09-14

**Authors:** LA Katargina, EV Denisova, AV Starikova, NV Lubimova, IP Nikishina

**Affiliations:** 1 Helmholtz Research Institute of Eye Diseases, Moscow, Russia; 2 Scientific Research Institute of Rheumatology of Russian Academy of Medical Sciences, Moscow, Russia

## Background

Despite various immunosuppressive agents, juvenile idiopathic arthritis (JIA) related uveitis often take a serious course that leads to sight threatening complications.

## Aim

To evaluate efficacy of Abatacept in combined treatment of JIA related uveitis.

## Methods

10 children aged 4 – 12 years with anterior uveitis associated with JIA were treated by Abatacept in standard infusions pattern and doses. Indication for using Abataceptwas ineffectiveness of standard therapy of arthritis and/or uveitis. 6 patients were suffering from polyarticular variants, 4 – oligoarticular JIA. Abatacept was combined with Methotrexate in 9, Sulfasalazin in 1 and low-dose steroids in 4 children. 3 patients were switched to Abatacept from Infliximab due to non-efficacy, 7 received abatacept as a first line biologic. 9 patients had bilateral eye involvement. At the administration of Abatacept severe inflammation was seen in 1, moderate in 7, mild in 2 of the cases. Follow up period ranged from 3 to 11 month (mean 7.6). The main outcome measure was the degree of inflammation.

## Results

Remission of uveitis in current treatment was achieved in 6 (60%), improvement in the degree of inflammation in 2 (20%) of the cases. The initial response was seen after the 2^nd^ – 4^th^ injection. From patients switched from Infliximab to Abatacept 1 achieved remission of uveitis, 1 improved, 1 remained stable. Patients with remission of uveitis diminished or discontinued topical medications. No ocular or systemic adverse effects were observed. Glaucoma or cataract surgery was uncomplicated in all 4 cases.

## Conclusions

Administration of Abatacept was effective in 80% of children with JIA related uveitis. Further investigations are required to define clear indications for this treatment in severe uveitis.

